# Increased mdr1 gene transcript levels in high-grade carcinoma of the bladder determined by quantitative PCR-based assay.

**DOI:** 10.1038/bjc.1994.130

**Published:** 1994-04

**Authors:** S. C. Clifford, D. J. Thomas, D. E. Neal, J. Lunec

**Affiliations:** Cancer Research Unit, Medical School, University of Newcastle-upon-Tyne, UK.

## Abstract

**Images:**


Br. J. Cancer (1994), 69, 680-686 © Macmillan Press Ltd., 1994

### Increased *mdr*1 gene transcript levels in high-grade carcinoma of the bladder determined by quantitative PCR-based assay

S.C. Clifford[1], D.J. Thomas[2], D.E. Neal[2] & J. Lunec[1]

[1]Cancer Research Unit, Medical School, and [2]Department of Surgery, William Leech Building, University of Newcastle-upon-Tyne, Newcastle-upon-Tyne NE2 4HH, UK.

**Summary** Overexpression of the multidrug resistance (*mdr*1) gene has been implicated in resistance to a number of the chemotherapeutic agents currently used in the treatment of bladder cancer (doxorubicin, vincristine and epirubicin). We report the development and validation of a quantitative assay for the determination of *mdr*1 gene transcript levels based on reverse transcription and the polymerase chain reaction (PCR), sensitive to less than 2-fold variations in transcript levels. Using these techniques, *mdr*1 mRNA levels were investigated in 32 primary untreated transitional cell carcinomas of the bladder. *mdr*1 mRNA was detected in all samples, with levels varying between individual tumours over a 63-fold range. These variations were not associated with the proliferative status of the tumour. *mdr*1 mRNA levels were significantly higher in poorly differentiated high-grade (G3) tumours than in well- and moderately differentiated low-grade (G1 and G2) tumours ($P = 0.0057$). The results suggest that this relationship may extend to *mdr*1 mRNA levels being an indicator of poor prognosis, as anticipated on the basis of the observed relationship to tumour stage and grade. No evidence was found to implicate *mdr*1 mRNA levels as a predictor of tumour recurrence or progression. Given that *mdr*1 mRNA levels are increased in a proportion of high-grade bladder tumours that are routinely subjected to chemotherapy, we discuss the possibility that *mdr*1 mRNA levels may be clinically significant as determinants of chemotherapeutic response and outcome in bladder cancer.

Cancer of the urinary bladder is the fifth most common malignancy in males of the western world, approximating to 16 new cases per 100,000 males per year in western populations (Davies, 1982). Transitional cell carcinomas (TCCs) of the urothelium constitute more than 90% of urothelial malignancies (Raghavan, 1988). Well-differentiated superficial tumours make up 60-65% of the bladder TCCs and may be treated by local resection with 5 year survival of 80% (Raghavan, 1988; Kiemeney *et al.*, 1993). However, the prognosis for patients presenting with muscle-invasive, dedifferentiated tumours is poor, with typical survival rates of 40-50% following combination radiotherapy and radical cystectomy (Skinner & Lieskovsky, 1984; Hendry, 1988).

Systemic chemotherapy is increasingly being used in the treatment of invasive bladder cancer, with combination regimens proving the most successful. Using an MVAC (methotrexate, vinblastine, doxorubicin, cisplatin) regimen, Sternberg *et al.* (1988) achieved a 67% response rate (37% complete) with median remission of over a year. Similarly, Harker *et al.* (1985) produced a 56% total (28% complete) response rate with median survival of 8 months using a CMV regimen. Both studies showed responses at all sites of the disease. However, it is clear that invasive tumours of the bladder are not wholly responsive to chemotherapy, with the majority of patients still dying of their disease. The reasons for this remain unclear - several cellular mechanisms have been described that may confer upon tumours resistance to many of the currently used chemotherapeutic drugs, of which the multidrug resistance (*mdr*1) gene has been the most widely studied to date.

Classical multidrug resistance is manifested by cross-resistance to a number of functionally unrelated lipophilic drugs of little structural similarity, including the vinca alkaloids (vincristine, vinblastine), the anthracyclines (doxorubicin, epirubicin), antibiotics (actinomycin D, mitomycin C) and taxol, and is thus implicated in resistance to a number of the agents currently used in the treatment of invasive bladder cancer. Acquisition of a multidrug resistance phenotype has been causally associated with expression of the *mdr*1 gene, which encodes P-glycoprotein (PGP), a 170 kDa plasma membrane protein that functions as an energy-dependent drug efflux pump resulting in decreased drug accumulation (Endicott & Ling, 1989; Van der Bliek & Borst, 1989). Cell line studies have shown good correlation between *mdr*1 mRNA levels and the degree of multidrug resistance (Shen *et al.*, 1986; Fojo *et al.*, 1987; Chan *et al.*, 1988; Noonan *et al.*, 1990).

*mdr*1 expression has been widely observed in many different human tumour and tissue types, with increased *mdr*1 levels frequently observed at relapse following chemotherapy (Fojo *et al.*, 1987; Goldstein *et al.*, 1989; Noonan *et al.*, 1990). Intrinsic variation in *mdr*1 expression levels may be an important determinant of tumour response, yet few studies have investigated variation of *mdr*1 mRNA levels within a specific tumour type prior to treatment, or related this to factors such as tumour grade and survival. Undetectable or low levels of *mdr*1 mRNA have been reported in both drug-sensitive and drug-resistant tumours prior to chemotherapy, lying close to or below the detection limits of conventional methods (Goldstein *et al.*, 1989; Noonan *et al.*, 1990). In bladder neoplasia, detection rates in untreated tumours have been mixed; at the protein level, Naito *et al.* (1992) reported detectable PGP in 32% of tumours by immunohistochemistry, while Benson *et al.* (1991) reported PGP expression in 71% of tumours using flow cytometry methods. At the message level, Goldstein *et al.* (1989) using Northern blot analysis reported only one weakly *mdr*1 mRNA-positive bladder tumour in six analysed. No previous studies have systematically investigated *mdr*1 mRNA expression in bladder cancer.

Assay insensitivity coupled with the often limited sample material available from tumours has hampered the detection of what may be clinically significant levels of *mdr*1 mRNA. To investigate whether such *mdr*1 mRNA levels lie below these detection limits, we report the development and validation of an assay based on reverse transcription and the polymerase chain reaction (PCR) for use in the determination of *mdr*1 mRNA levels in clinical samples. Using these methods we have determined the incidence of and variation in *mdr*1 transcript levels in a series of untreated TCCs of the bladder, and investigated the relationship of these levels to tumour stage, grade and rate of proliferation, prognosis, survival, progression and recurrence.

Correspondence: J. Lunec.
Received 5 August 1993; and in revised form 22 November 1993.

### Materials and methods

*mdr*l transcript levels were measured relative to those of 18S ribosomal RNA as an internal reference. The MHC class II related protein, $\beta_2$-microglobulin ($\beta_2$-M), has previously been used as an internal reference for the determination of *mdr*l transcript levels by polymerase chain reaction (PCR)-based methods (Kuwazuru *et al.*, 1990; Noonan *et al.*, 1990). We have also investigated the suitability of $\beta_2$-M mRNA levels as an internal reference for the measurement of *mdr*l mRNA levels in bladder cancer.

### *Tumours and tissues*

TCC tumour samples obtained at resection or cystectomy were immediately snap frozen in liquid nitrogen and transferred to a $-80$°C freezer for storage. A portion of the sample was sent for histological assessment and the tumours were staged and graded according to UICC (1978) criteria. All samples described in this study had not received prior chemotherapy. Eleven out of 18 of the patients with invasive tumours (T2–T4) went on to undergo chemotherapy. Adrenal tissue was obtained from patients undergoing radical nephrectomy.

Care was taken to limit the proportion of the tumour sample contaminated by normal tissue. Because of their papillary growth pattern superficial tumours were readily removed without contamination from underlying normal tissue. Although the invasive tumours were more difficult to separate from the underlying lamina propria and muscle, contamination by normal tissue was minimised by only taking samples from the protruding mass of the tumour. Although it was difficult to ensure complete elimination of normal tissue from these samples, histological examination indicated an upper limit of 10–15% normal tissue.

### *Cell lines*

Multidrug-resistant cell lines known to overexpress the *mdr*l gene and their parental controls were used for assay development and validation. All lines were tested and found to be mycoplasma negative.

The KK47 cell line was established from an untreated grade 1, stage Ta TCC of the bladder, from which the multidrug-resistant cell line KK47/ADM was derived by stepwise selection in increasing concentrations of doxorubicin (Kimiya *et al.*, 1992). Both lines were grown as monolayer cultures in complete minimum essential medium (MEM) at 37°C in a 5% carbon dioxide atmosphere, and subcultured weekly by harvesting with a 0.25% trypsin – 0.02% EDTA solution and reseeding at $3 \times 10^6$ cells per 175 cm$^2$ flask.

The small-cell lung cancer (SCLC) cell line NC1-H69P was derived from a patient who had previously received multidrug therapy. Its multidrug-resistant variant, H69/LX4, was derived by stepwise selection in doxorubicin (Twentyman *et al.*, 1986). Both lines were cultured in RPMI-1640 medium (Dutch modification) (Northumbria Biochemicals) supplemented with 10% fetal calf serum, L-glutamine (2 mM), penicillin (100 U ml$^{-1}$) and streptomycin (100 $\mu$g ml$^{-1}$) at 37°C in a 5% carbon dioxide atmosphere. Doxorubicin (0.4 $\mu$g ml$^{-1}$) was added to H69/LX4 cultures. Both lines were grown as non-adherent batch cultures, and resuspended in fresh medium every 4 days.

### *RNA extraction*

Total RNA was extracted from the remaining tumour tissue and cell lines using the RNAzol method (Chomzynski & Sacchi, 1987). Briefly, in the case of solid tumours, tissue was pulverised in liquid nitrogen and rapidly lysed in a phenol–guanidinium thiocyanate–$\beta$-mercaptoethanol mixture. Cell lines were harvested at 70% confluence by scraping in ice-cold phosphate-buffered saline (PBS) (KK47 and KK47/ADM) or by centrifugation followed by washing in PBS (H69 and H69/LX4). Both methods preceded direct lysis as described. RNA was isolated by chloroform extraction and precipitated by isopropanol addition. Following washing in ethanol and resuspension in sterile water, RNA yield and purity were checked by spectrophotometric determination at 260 nm and 280 nm and the integrity assessed by electrophoretic size separation on 1.4% agarose gels.

### *cDNA synthesis*

First-strand cDNA was synthesised from total RNA by reverse transcription using the random primer extension method. A 10 $\mu$g aliquot of total RNA was heated to 100°C for 5 min, then added to a 200 $\mu$l reaction (final volume) consisting of the following: pd(N)$_6$ random hexamers (0.88 units; Pharmacia), dithiothreitol (0.01 M; Gibco, BRL), dATP, dCTP, dTTP and dGTP (1 mM each; Pharmacia), Moloney murine leukaemia virus reverse transcriptase (1200 units, Gibco BRL) and human placental RNAse inhibitor (35 units; Gibco BRL) made up in 1 × reverse transcriptase buffer (Gibco BRL). The reaction was allowed to proceed at 37°C for 1.5 h before termination by heating to 100°C for a further 5 min. Following synthesis, cDNA was purified by passage through a cDNA spun column containing Sephacryl S-300 (Pharmacia) to remove any remaining proteins, free nucleotides and random hexamers remaining from previous procedures. The second cDNA strand was synthesised by the extension of sequence-specific oligonucleotide primers in the first cycle of a subsequent polymerase chain reaction.

### *Optimisation of the polymerase chain reaction*

*mdr*l and $\beta_2$-M primers chosen were those used by Noonan *et al.* (1990). The *mdr*l primers distinguish between the *mdr*l and *mdr*2 gene sequences, and both the *mdr*l and $\beta_2$-M primers span an intron to control against contamination by amplification of genomic DNA sequences. Primers to 18S rRNA were chosen using a computer program designed by Lowe *et al.* (1990) – ATGCTCTTAGCTGAGTGTCC and AACTACGACGGTATCTGATC (residues 763–782 and 1055–1074 respectively; Gonzalez & Schmickel, 1986). Primers were made on an oligonucleotide synthesiser (Model 392, Applied Biosystems). The primers yielded products of 167 base pairs (*mdr*l), 120 base pairs ($\beta_2$-M) and 311 base pairs (18S rRNA). For each set of primers, a series of fixed-condition PCR reactions were performed employing a fixed concentration of input cDNA. The absolute magnesium chloride concentration was varied in 0.5 mM steps in the 0–5 mM range. The optimal magnesium chloride concentrations were determined to be: *mdr*l, 2.5 mM; $\beta_2$-M, 3 mM; and 18S rRNA, 1 mM.

### *Determination of* mdrl *mRNA levels*

Typical PCR reaction yields for various cDNA inputs are shown in Figure 1. An initial range-finding experiment was performed to determine the range of serial cDNA dilutions over which PCR amplification is linear for each target species. Serial cDNA dilutions for each species are simultaneously and independently amplified over 25 PCR cycles (94°C for 1 min, 56°C for 1 min, 72°C for 1 min) using otherwise fixed reaction conditions. A 25 $\mu$l reaction consisted of the following (final concentrations are stated): 10 $\mu$l of appropriate cDNA dilution, *Taq* polymerase (0.75 units; Advanced Biotechnologies), dNTPs (0.25 mM dTTP, dCTP and dGTP, 0.125 mM dATP; Pharmacia), 0.25 $\mu$l of [$\alpha$-$^{32}$P]dATP (Amersham), oligonucleotide primers (0.48 $\mu$M each) and the magnesium chloride component (2.5 mM *mdr*l, 3 mM $\beta_2$-M and 1 mM 18S rRNA; Advanced Biotechnologies), made up in a final concentration of 1 × PCR reaction buffer (Advanced Biotechnologies), and overlaid with mineral oil. Following amplification, 10 $\mu$l aliquots were analysed by electrophoretic separation on 12% polyacrylamide gels at 100 V for 1.5 h. Gels were dried under heat and vacuum and the radioactively labelled PCR products detected and analysed using a PhosphorImager (Molecular Dynamics). A typical autoradiograph image of the separated products is shown in Figure 1. For each species, the amount of PCR product (measured as incorporated radioactivity) was plotted against input cDNA dilution (see Figure 2). Regression analysis was performed on the points constituting the linear range of amplification for each species. The ratio of input total cDNA concentrations ($X_1/X_2$ in Figure 3) for a given yield in the linear range of amplification is a measure of the ratio of cDNA concentrations present for each species. This is equal to the slope ratio ($M_1/M_2$) for the regression lines on the linear part of the yield curve (see Figure 3). Thus, *mdr*l mRNA levels were expressed as an *mdr*l/18S ratio. Subsequent replicates were performed using only four serial dilutions (lying within the predefined linear amplification ranges) per species. Analysis was performed in triplicate on all samples. A linear regression correlation coefficient ($r^2$) of greater than 0.95 was used as the criterion for accepting data that fell within the linear range of amplification.

*Determination of tumour proliferative status – MIB1 analysis*

The monoclonal antibody MIB1 reacts with the human nuclear cell proliferation-associated antigen recognised by the monoclonal antibody, Ki67, that is expressed in all active parts of the cell cycle (Cattoretti *et al*., 1992). The extent of MIB1 staining was thus used to assess the proliferative status of a tissue.

Paraffin sections (4 µm thick) were taken and dewaxed in xylene prior to rehydration through alcohol and water. Sections were treated with 0.1 M citrate buffer (pH 6.0) and microwaved for 20 min. Endogenous peroxidase activity was blocked by prior treatment with 3% hydrogen peroxide. The sections were then rinsed in Tris-buffered saline (TBS pH 7.6)

**Figure 1** Products produced from simultaneous independent amplification over 25 PCR cycles of serial cDNA dilutions derived from a bladder tumour sample for *mdr*l, $\beta_2$-M and 18S, following separation by polyacrylamide electrophoresis and visualisation by autoradiography.

**Figure 2** Graph showing a log–log plot of product produced versus initial amount of input cDNA for the three series of points shown in Figure 1. The linear ranges of amplification used in subsequent quantifications are highlighted for each species by black arrows. Note that these ranges lie between the ranges of reaction threshold and saturation. Δ, *mdr*l; ●, $\beta_2$-M; O, 18S.

for 2 min, followed by normal goat serum (Life Science). Sections were incubated with MIB1 antibody (The Binding Site) (diluted 1:50) for 60 min, washed again and peroxidase activity developed using biotinylated goat anti-mouse/rabbit secondary antigen (Dako) for 30 min. After further washing in TBS, sections were incubated in streptavidin AB complex/horseradish peroxidase (Dako) for 30 min. Sections were then incubated in diaminobenzidine solution for 10 min, washed and counterstained in Carruzi's hamatoxylin, dehydrated and mounted. A negative control was performed for each tissue section by omission of primary antiserum. Tonsillar tissue was used as a positive control. The proportion of tumour cell nuclei staining with MIB1 was assessed in random fields from well-preserved areas of tumour. A minimum of 2,000 cells were counted in each case. Nuclei in morphologically malignant cells were considered positive when dark-brown nuclear staining was observed.

### Results

*Assay validation*

For any given tumour RNA sample, the standard error of the mean based on the repeat experiments was typically ± 23% of the mean value. Experiments performed on the H69/LX4 cell line indicate that three separate analyses of a sample utilising independent cell cultures, RNA extractions and cDNA syntheses produces results (*mdr*l/18S ratios) lying within a range of $1.807 \pm 0.911 \times 10^{-3}$ (mean ± s.d.).

To examine any influence that PCR amplification efficiency may have on our results, efficiency studies were performed on four different tissue types with varying *mdr*l/18S levels. Figure 4a and b shows the results for a typical bladder tumour RNA sample as an example. Reaction efficiency was calculated using the formula $N_n = N_{n-1}\ (1 + \varepsilon)$, where $N_n$ is the amount of product after the *n*th cycle, *n* is the cycle number and ε is the efficiency. 18S rRNA was amplified with the greatest efficiency, followed by *mdr*l then $\beta_2$-M (Figure 4b). This order of efficiency remained constant for tissues analysed. For each product species, the absolute amplification efficiency showed some variation between samples, however relative amplification efficiencies remained consistent between samples, irrespective of tissue type or level of *mdr*l expression (Table I). The maximum efficiency range corresponds to the linear range of amplification, and its position is dependent on the input cDNA concentration. The assay that we have described utilises cDNA concentrations in the range that gives linear amplification over 25 PCR cycles.

**Figure 3** Illustration showing the calculation of relative mRNA levels for two gene product species. Lines labelled 'Gene products 1 and 2' represent the linear ranges of amplification for two given species, extrapolated through the origin. In the linear amplification range, the ratio of input cDNA for a given product ($X_1/X_2$) is a measure of the relative amounts of mRNA for the two genes under consideration. For common extrapolation through zero, this is equal to the slope ratio ($M_1/M_2$) for the two lines.

utilises cDNA concentrations in the range that gives linear amplification over 25 PCR cycles.

### $\beta_2$-*Microglobulin as an internal reference*

$\beta_2$-M mRNA levels in individual bladder tumours were found to vary over a 140-fold range when measured relative to 18S rRNA. Similarly, *mdr*l mRNA levels measured relative to $\beta_2$-M did not correlate with those measured relative to 18S rRNA ($r^2 = 0.017$). Thus $\beta_2$-M transcript levels vary widely between bladder tumour samples and were therefore considered unsuitable as an internal reference. For subsequent analyses *mdr*l mRNA levels were expressed as ratios relative to 18S rRNA.

### mdr*1 mRNA levels in TCCs of the bladder*

*mdr*l mRNA was detected in all bladder tumour samples analysed (Figure 5) ($n = 32$; by stage, Ta, $n = 9$; T1, $n = 6$; T2, $n = 2$; T3, $n = 9$; T4, $n = 6$; by grade, G1, $n = 2$; G2, $n$ 12; G3, $n = 18$), with a mean *mdr*l/18S ratio of $7.34 \times 10^{-6}$. A 63-fold variation in *mdr*l/18S levels was observed between individual tumours (highest $3.4 \times 10^{-5}$; lowest = $5.4 \times 10^{-7}$).

**Figure 4** **a**, A PCR efficiency study performed on a bladder tumour sample (*mdr*l/18S ratio = $3.4 \times 10^{-6}$). *mdr*l ($\Delta$, $10^0$ cDNA dilution), $\beta_2$-M (●, $10^{-1}$ dilution) and 18S (O, $10^{-4}$ dilution) were amplified over 33 PCR cycles and product determined at alternate cycles between cycles 15 and 33. Product is shown plotted against cycle number (linear scale). Note the typical PCR reaction kinetics of (1) a detection threshold in the early cycles, (2) a linear range of amplification and (3) a reaction saturation plateau in the later cycles. **b**, Changes in reaction efficiency over subsequent PCR cycles for the analysis shown in **a**. For each product species, efficiency (1 = 100%) as defined in the text is plotted against cycle number. Graphs produced show efficiency rising to a peak that corresponds to the linear phase of amplification, then tailing off as the reaction reaches saturation.

### *Relationship of* mdr*1 mRNA levels to stage and grade*

*mdr*l/18S ratios were significantly higher in poorly differentiated high-grade (G3) than in well- and moderately differentiated low-grade (G1 and 2) tumours (Figure 5). The pooled *mdr*l/18S mean ± s.e. for grades G1 and G2 combined was $3.41 \pm 0.53 \times 10^{-6}$ compared with $10.40 \pm 2.2 \times 10^{-6}$ for the G3 tumours (*t*-test, $P = 0.0057$). No low-grade tumours showed high *mdr*l levels, and while not all high-grade tumours showed elevated *mdr*l/18S ratios there was a markedly increased incidence of high-expressing tumours in this group (Figure 5). With the exception of a single histologically atypical tumour which presented as T1 G3 (carcinoma *in situ*), all superficial tumours (Ta and T1) were also low grade, while all invasive tumours (T2, T3 and T4) were high grade. Thus, the *mdr*l expression pattern observed for tumour grade essentially extends to tumour stage; based on pooled means, *mdr*l/18S ratios were marginally significantly higher ($P = 0.085$) in muscle-invasive tumours than in superficial, non-invasive tumours. Removal of the atypical carcinoma *in situ* sample from the analysis increases the level of significance ($P = 0.012$). The two normal tissue samples examined showed higher levels of *mdr*l mRNA than the superficial tumour group and the levels were similar to those seen in the high-grade invasive tumours.

### *Relationship between* mdr*1 expression and progression, recurrence and survival*

For these analyses, tumours were split into high-*mdr*l mRNA level (*mdr*l/18S ratio $> 1 \times 10^{-5}$, $n = 8$) and low-*mdr*l mRNA level (*mdr*l/18S ratio $< 9.99 \times 10^{-6}$, $n = 24$) groups. This distinction was made on the basis of the distribution of expression observed, with all high-*mdr*l mRNA tumours lying outside the main cluster of points.

No evidence was found to implicate *mdr*l mRNA levels as a predictor of tumour recurrence or progression. No correlation ($r = 0.08$, $n = 13$) existed between *mdr*l/18S ratios and the rate of recurrence. Forty-three per cent (three of seven) high-*mdr*l-expressing tumours underwent progression, compared with 33% (4 of 12) of low-*mdr*l-expressing tumours. There was no significant difference between these incidences of progression (Fisher's exact test, $P = 0.35$). Of patients with tumours with low *mdr*l mRNA levels, 64% (14 of 22) were still alive at the end of their current follow-up period, compared with 25% (2 of 8) of high-*mdr*l mRNA-expressing tumours. The minimum follow-up period was 32 months, with all deaths occurring within 72 months of biopsy. Only patients dying from their disease were included in the analysis, regardless of treatment received. Log-rank survival analysis (Peto *et al.*, 1977) showed no significant difference ($P = 0.36$) between survival for the groups of tumours with high and low *mdr*l mRNA levels (Figure 6). However, contingency table analysis using Fisher's exact test showed a lower proportion of survivors for patients with high *mdr*l

**Table I** The results of amplification studies on the four different tissues. Relative amplification efficiencies between product species are shown (NA = results not available)

| | | PCR amplification efficiency ratios | | |
|---|---|---|---|---|
| *Tissue type* | *mdr1/18S Ratio* | *mdr1/$\beta_2$-M* | *mdr1/18S* | *$\beta_2$-M/18S* |
| H69/LX4 | $2.8 \times 10^{-3}$ | 1.14 | 0.875 | 0.77 |
| Adrenal | $2 \times 10^{-4}$ | 1.19 | NA | NA |
| Normal bladder | $3.9 \times 10^{-5}$ | 1.16 | 0.88 | 0.75 |
| Bladder tumour | $3.4 \times 10^{-6}$ | NA | 0.94 | NA |

**Figure 5** Individual tumour *mdr*l mRNA levels measured relative to 18S rRNA [shown as mean *mdr*l/18S ratio (± standard error), $n \geq 3$]. Tumours are shown grouped by stage of progression and the histological grade is indicated by shading: open, normal bladder; single-hatched, G1; cross-hatched, G2; solid, G3. Note the increased incidence of higher expressing tumours in the high-grade group.

**Figure 6** Log-rank survival curves for groups of patients with tumours expressing high and low *mdr*l mRNA levels. Each death is represented by a vertical drop on the graph. Surviving patients are shown as a vertical tick on the graph representing the end of their current follow-up period. The log-rank test gave $P = 0.36$ for no difference in survival rates between the low- (---) and high-*mdr*l (——) mRNA groups (see also text).

mRNA tumours, which was marginally significant $P = 0.07$).

*Relationship between* mdr*l mRNA levels and tumour proliferative status*

The percentage of proliferating cells within the tumours studied (those displaying the Ki67 antigen) did not correlate with their *mdr*l/18S ratios ($r = 0.06$, $n = 24$).

*Other* mdr*l mRNA determinations*

As part of general method validation and to put our data into a broader context, *mdr*l/18S ratios were also determined for some other tissues and cell lines with known low and high levels of *mdr*l expression. Adrenal tissue had an *mdr*l mRNA level (*mdr*l/18S = $2.00 \times 10^{-4}$) approximately 27 times higher than the mean level of expression in bladder tumours. Hyperexpression ($1.58 \times 10^{-3}$) was detected in the *mdr* SCLC cell line H69/LX4, while no *mdr*l expression could be quantified in its drug-sensitive parent line H69 at 25 PCR cycles. The *mdr* bladder tumour cell line KK47/ADM had 157-fold overexpressed *mdr*l mRNA levels (*mdr*l/18S ratio $3.88 \times 10^{-4}$), compared with levels of $2.46 \times 10^{-6}$ in its parental line KK47.

### Discussion

*The PCR-based transcription assay*

Measurement of expression relative to an endogenous internal reference avoids the need for addition and quantification/titration of an external reference standard, or the manipulation of products post amplification to distinguish them (e.g. the presence of restriction enzyme cleavage site in a synthetic reference standard), both strategies being among those previously described (Becker-Andre & Hahlbrock, 1989; Wang *et al.*, 1989). 18S rRNA is widely used to control for equal loading and transfer in Northern blot methods for RNA analysis. Good evidence exists for the use of rRNA (of which 18S rRNA is one component) as a reference. While variations in total rRNA levels per cell do occur, these are usually accompanied by an equal fluctuation in total mRNA levels (Johnson *et al.*, 1976). Hirsch (1967) observed consistent rRNA levels (80% of total) in normal rat liver, fasted rat liver (with a 50% reduction in total RNA content) and rapidly dividing rat hepatoma. $\beta_2$-Microglobulin has been commonly used as an internal reference for the determination of *mdr*l transcript levels in PCR-based studies (Kuwazuru *et al.*, 1990; Noonan *et al.*, 1990). However, our studies have demonstrated a 140-fold variation in $\beta_2$-M mRNA levels between individual bladder tumours, rendering it completely unacceptable for such a purpose. Whether these observations extend to other tumour systems remains to be investigated. Amplification of target and reference from a common cDNA source controls for variations in efficiency of both RNA extraction and cDNA synthesis. Our efficiency studies indicate that, while absolute amplification efficiencies vary between different species, their relative efficiencies remain constant regardless of tissue type or *mdr*l/18S ratio and thus have no influence on the results observed.

Based on the typical standard error of 23% of the mean of three replicates, this assay is capable of discriminating 1.6-fold differences in mRNA levels between samples at the 95% confidence level. The reproducibility studies based on three independent batch cultures, RNA extractions and cDNA syntheses similarly indicate that *mdr*l mRNA levels from three independent analyses of a given sample fall within a 2.5-fold range, however this may overestimate the situation since the batch cultures were not harvested at uniform cell densities and may also be prone to differing culture conditions, both of which may affect cellular mRNA levels. These limits are small compared with the 63-fold variation that we have observed in *mdr*l mRNA levels between individual tumour samples.

For the assay as described, 10 μg of total RNA yields sufficient cDNA to perform at least five replicates of the assay, although there are various ways in which the sensitivity could be further increased if necessary. In comparison, Northern blot analysis may require 10–20 μg of RNA for a single analysis. In the clinical situation, in which sample material is often limited, this may not allow for a repeat analysis. This method offers notable improvements in sensitivity over conventional methods, detecting *mdr*l transcript in all samples analysed, whereas previous studies using Northern blot analysis have only detected *mdr*l mRNA positivity in one of six (<17%) bladder tumours (Goldstein *et al.*, 1989). In this context, categorisation of samples as *mdr*l mRNA positive/negative is misleading since the distinction is made by the arbitrary detection threshold of a particular technique rather than the position of the data within a distribution.

As with Northern blot methods, the heterogeneous nature of tumours is one potential problem in this type of analysis. The assay provides an overall mean mRNA level for the whole sample analysed. It is therefore essential that the tumour sample used is representative of the entire sample.

To test the ability of the assay to detect differences in *mdr*l mRNA levels, we examined adrenal gland as an example of a high-expressing tissue, and also multidrug-resistant lung and bladder tumour cell lines known to overexpress P-glycoprotein. Adrenal gland tissue has been widely reported to have high levels of *mdr*l mRNA (Fojo *et al.*, 1987; Noonan *et al.*, 1990). Noonan *et al.* (1990) reported a single bladder tumour to have *mdr*l mRNA levels 68 times lower than those in adrenal tissue. This is in agreement with our study, which shows the mean bladder tumour *mdr*l/18S ratio to be 27 times lower than that of an adrenal sample. Likewise, the *mdr*l/18S ratio for the KK47 cell line, derived from a superficial bladder tumour, lies in the same range as all the superficial bladder tumour samples analysed.

We have thus developed a highly sensitive and reproducibly accurate PCR-based assay for the quantification of low-level gene expression. Should it be necessary, sensitivity of the assay could be even further improved with increased numbers of PCR amplification cycles. Further replicates would also increase confidence in the mean for a given sample. This technique may be applied to the quantification of any low-level gene expression when the cDNA sequence is known, with the accurate quantification of results allowing a much more detailed analysis of the data. The assay could potentially be adapted to work at the DNA level for the determination of gene copy numbers and levels of gene amplification.

### *Variation in* mdr*l mRNA levels in TCC of the bladder*

*mdr*l/18S ratios were found to vary over a 63-fold range in bladder tumours. The mechanisms underlying this remain to be elucidated. The lack of any correlation between *mdr*l/18S ratios and tumour Ki67 levels indicates that the mechanism underlying variation in tumour *mdr*l mRNA levels is not simply a result of tumour proliferation or the rate of cell turnover. Similarly, since these tumours have not been subjected to selection by chemotherapy, it seems unlikely that gene amplification plays any role in the variation of *mdr*l mRNA levels observed. Transcriptional control mechanisms have been suggested to play a significant role in the regulation of *mdr*l mRNA levels (Goldstein *et al.*, 1989; Zastawny *et al.*, 1993). Recently it has been demonstrated that wild-type p53 protein represses human *mdr*l promoter activity while mutant forms (cysteine 135 to serine) of the p53 protein enhance *mdr*l transcription (Zastawny *et al.*, 1993), thus defining a possible mechanism for increased *mdr*l mRNA levels in high-grade untreated bladder tumours, in which frequent p53 alterations have been reported (Lunec & Mellon, 1994). The stage and grade associations that we have observed suggest that overexpression could also be a result of the loss of genetic regulation and control associated with high-grade (dedifferentiated) tumours. The level of *mdr*l mRNA detected in normal bladder raised questions about the contribution of normal tissue contamination to the increased levels of *mdr*l mRNA found in the high-grade tumours compared with the superficial group. However, because of the careful resection technique, the maximum observed contamination of the invasive tumours by normal tissue of only 10–15% could not have accounted for the elevated levels of *mdr*l mRNA seen in the high-grade group.

### *Relationships between* mdr*l mRNA levels and prognosis/survival*

Our results demonstrate that *mdr*l mRNA levels are clearly elevated in high-grade bladder tumours. A recent immunohistochemistry study also showed increased levels of the *mdr*l gene product, P-glycoprotein, in high-stage neuroblastomas (Chan *et al.*, 1991). Thus, evidence exists for *mdr*l mRNA and P-glycoprotein levels being a marker of tumour aggression (invasiveness and dedifferentiation). However, the majority of studies are conducted at the protein level, with this study to our knowledge being the first to report such an association at the mRNA level. Other studies investigating P-glycoprotein levels in bladder cancer (Benson *et al.*, 1991; Naito *et al.*, 1992) have proved inconclusive, showing no clear relationships with stage and grade.

The population distribution of *mdr*l/18S ratios observed in bladder tumours has allowed us to distinguish groups of high- and low-expressing tumours for use in survival analysis. Log-rank tests showed no significant difference in survival for either group of tumours (Figure 6), however this analysis is complicated by the problem of non-uniform patient treatment and follow-up times. The analysis by Fisher's exact test based on proportions of survivors alone indicates a significant relationship between high *mdr*l mRNA levels and poor survival. Other studies have similarly reported high *mdr*l expression to be an indicator of adverse prognosis and poor survival in untreated tumours; at the protein level in soft-tissue sarcomas (Chan *et al.*, 1990), breast carcinoma (Verrelle *et al.*, 1991), neuroblastoma (Chan *et al.*, 1991) and non-lymphoblastic leukaemia (Campos *et al.*, 1992), and at the message level in acute myeloid leukaemia (Pirker *et al.*, 1991). Our results suggest that it may be worth investigating further the prognostic potential of *mdr*l mRNA levels in bladder cancer. It may be interesting to compare *mdr*l mRNA with other known prognostic factors using samples from patient groups undergoing either uniform treatment strategies or receiving no treatment.

We have demonstrated that *mdr*l mRNA levels are raised in many of the high-grade, invasive bladder tumours that are commonly treated using chemotherapy, and suggest that the 34-fold variation in *mdr*l mRNA levels observed between individual high-grade tumours could be a significant determinant of their chemotherapeutic response. This is supported by *in vitro* studies which show that even small variations in *mdr*l mRNA levels, within the range we have observed, result in significant differences in drug response (Noonan *et al.*, 1990). To evaluate the role of pretreatment *mdr*l mRNA levels in the determination of chemotherapeutic outcome, full prospective studies have been initiated involving uniform treatment regimens with pre- and post-chemotherapy tumour biopsies. This will also test whether *mdr*l mRNA levels are increased following treatment, as has been reported in some tumour systems (Fojo *et al.*, 1987; Noonan *et al.*, 1990).

Many thanks to Dr S. Freemantle for her part in the development of PCR methods. The cell lines used as controls in this study were kindly supplied by Dr Peter Twentyman (H69 and H69/LX4) and Dr Seiji Naito (KK47 and KK47/ADM). This work was generously funded by the North of England Cancer Research Campaign.

### References

BECKER-ANDRE, M. & HAHLBROCK, K. (1989). Absolute mRNA quantitation using the polymerase chain reaction (PCR). A novel approach by PCR aided transcript titration assay (PATTY). *Nucleic Acids Res.*, **17**, 9437-9466.

BENSON, M.C., GIELLA, J., WHANG, I.S., BUTTYAN, R., HENSLE, T.W., KARP, F., & OLSSON, C.A. (1991). Flow cytometric determination of the multidrug resistant phenotype in transitional cell cancer of the bladder: implications and applications. *J. Urol.*, **146**, 982-987.

CAMPOS, L., GUYOTAT, D., ARCHIMBAUD, E., CALMAR-ORIOL, P., TSURO, T., TRONCY, J., TRIELLE, D. & FIERRE, D. (1992). Clinical significance of multidrug resistance P-glycoprotein expression in acute nonlymphoblastic leukaemia cells at diagnosis. *Blood*, **79**, 473-476.

CATTORETTI, G., BECKER, M.H.G., KEY, G., DUCHROW, M., SCHLUTER, C., GALLE, J. & GERDES, J. (1992). Monoclonal antibodies against recombinant parts of the Ki-67 antigen (MIB1 and MIB3) detect proliferating cells in microwave-processed paraffin sections. *J. Pathol.*, **168**, 357-363.

CHAN, H.S., BRADLEY, G., THORNER, P., HADDAD, G., GALLIE, B.L. & LING, V. (1988). A sensitive method for immunocytochemical detection of P-glycoprotein in multidrug resistant human ovarian carcinoma cell lines. *Lab. Invest.*, **59**, 870-875.

CHAN, H.S., THORNER, P.S., HADDAD, G. & LING, V. (1990). Immunohistochemical detection of P-glycoprotein: prognostic correlation in soft tissue sarcoma of childhood. *J. Clin. Oncol.*, **8**, 689-704.

CHAN, H.S., HADDAD, G., THORNER, P.S., DEBOER, G., LIN, Y.P., ONDRUSEK, N., YEGER, H. & LING, V. (1991). P-glycoprotein expression as a predictor of the outcome of therapy for neuroblastoma. *N. Engl. J. Med.*, **325**, 1608-1614.

CHOMZYNSKI, P. & SACCHI, N. (1987). Single step method of RNA isolation by acid guanidium thiocyanate phenol chloroform extraction. *Anal. Biochem.*, **162**, 156-159.

DAVIES, J.M. (1982). Occupational and environmental factors in bladder cancer. In *Scientific Foundations of Urology*, 2nd edn, Chisholm, G.D. & Innes-Williams, D. (eds), pp. 732-737. Heineman: London.

ENDICOTT, J.A. & LING, V. (1989). The biochemistry of P-glycoprotein mediated resistance. *Annu. Rev. Biochem.*, **58**, 136-171.

FOJO, J.A., UEDA, K., SALMON, D.J., POPLACK, D.G., GOTTESMAN, M.M. & PASTAN, I. (1987). Expression of a multidrug resistance gene in human tumors and tissues. *Proc. Natl Acad. Sci. USA*, **84**, 265-269.

GOLDSTEIN, L.J., GALSKI, H., FOJO, A., WILLINGHAM, M., LAI, S.-L., GAZDAR, A., PIRKER, R., GREEN, A., CRIST, W., BRODEUR, G.M., LIEBER, M., COSSMAN, J., GOTTESMAN, M.M. & PASTAN, I. (1989). Expression of a human multidrug resistance gene in human cancers. *J. Natl. Cancer Inst.*, **81**, 116-124.

GONZALEZ, I.L. & SCMICKEL, R.D. (1986). The human 18S ribosomal RNA gene: evolution and stability. *Am. J. Hum. Genet.*, **38**, 419-427.

HARKER, W.G., MEYERS, F.J., FREIHA, F.S., PALMER, J.M., SHORTLIFFE, L.D., HANNIGAN, J.F., MCWHIRTER, K.M. & TORTI, F.M. (1985). Cisplatin, methotrexate and vinblastine (CMV): An effective chemotherapy regimen for metastatic transitional cell carcinoma of the urinary tract. A Northern California Oncology Group study. *J. Clin. Oncol.*, **3**, 1463-1470.

HENDRY, W.F. (1988). Diagnosis and management of bladder cancer: a British perspective. In *The Management of Bladder Cancer*, Raghavan, D. (ed.), pp. 67-93. E.J. Arnold: London.

HIRSCH, C.A. (1967). Quantitative determination of the ribosomal ribonucleic acid content of liver and Novikoff hepatoma from fed and fasted rats. *J. Biol. Chem.*, **242**, 2822-2827.

JOHNSON, L.F., ABLESON, H.T., PENMAN, S. & GREEN, H. (1976). The relative amounts of cytoplasmic RNA species in normal, transformed and senescent cultured cell-lines. *J. Cell Physiol.*, **90**, 465-470.

KIEMENEY, L.A.L.M., WITJES, J.A., VERBEEK, A.L.M., HEIJBROEK, R.P., DEBRUYNE, F.M.J. & THE MEMBERS OF THE DUTCH SOUTH-EAST COOPERATIVE UROLOGICAL GROUP. (1993). The clinical epidemiology of superficial bladder cancer. *Br. J. Cancer*, **67**, 806-812.

KIMIYA, K., NAITO, S., SOEJIMA, T., SAKAMOTO, M., KOTOH, S., KUMAZAWA, J. & TSURO, T. (1992). Establishment and characterisation of doxorubicin resistant human bladder cell line, KK47/ADM. *J. Urol.*, **148**, 441-445.

KUWAZURU, Y., YOSHIMURA, A., HANADA, S., UTSUNOMIA, A., MAKINO, T., ISHIBASHI, K., KODOMA, M., IWAHASHI, M., ARIMA, T. & AKIYAMA, S.-I. (1990). Expression of the multidrug transporter, P-glycoprotein, in acute leukaemia cells and correlation to clinical resistance. *Cancer*, **66**, 868-873.

LOWE, T., SHREFKIN, J., YANG, S.Q. & DIFFENBACH, C.W. (1990). A computer program for selection of oligonucleotide primers for polymerase chain reactions. *Nucleic Acids Res.*, **18**, 1757-1761.

LUNEC, J. & MELLON, K. (1994). Molecular Biology and Bladder Cancer. In *Frontiers in Uro-Oncology*. Neal, D.E. (ed.). Springer (in press).

NAITO, S., SAKAMOTO, N., KOTOH, S., GOTO, K., MATSUMOTO, T. & KUMAZAWA, J. (1992). Correlation between the expression of P-glycoprotein and multidrug-resistant phenotype in transitional cell carcinoma of the urinary tract. *Eur. Urol.*, **22**, 158-162.

NOONAN, K.E., BECK, C., HOLZMAYER, T.A., CHIN, J.E., WUNDER, J.S., ANDRULIS, I.L., GAZDAR, A.F., WILLMAN, C.L., GRIFFITH, B., VON HOFF, D.D. & RONINSON, I.B. (1990). Quantitative analysis of *mdr*1 gene expression in human tumors by polymerase chain reaction. *Proc. Natl Acad. Sci. USA*, **87**, 7160-7164.

PETO, R., PIKE, M.C., ARMITAGE, P., BRESLOW, N.E., COX, D.R., HOWARD, S.V., MANTEL, N., MCPHERSON, K., PETO, J. & SMITH, P.G. (1977). Design and analysis of randomized clinical trials requiring prolonged observation of each patient: II Analysis and examples. *Br. J. Cancer*, **35**, 1-39.

PIRKER, R., WALLNER, J., GEISSLER, K., LINKENSCH, W., HAAS, O.A., BETTELHEIM, P., HOPFNER, M., SCHERRER, R., VALENT, P., HAVALEC, L., LUDWIG, H. & LECHNER, K. (1991). *mdr*1 gene expression and treatment outcome in acute myeloid leukaemia. *J. Natl Cancer Inst.*, **83**, 708-712.

RAGHAVAN, D. (1988). The management of bladder cancer: current practice and future prospects. In *The Management of Bladder Cancer*, Raghavan, D. (ed.), pp. 317-332. E.J. Arnold: London.

SHEN, D.W., FOJO, A., RONINSON, I.B., CHIN, J.E., SOFFIR, R., PASTAN, I. & GOTTESMAN, M.M. (1986). Multidrug resistance of DNA-mediated transformants is linked to transfer of the human *mdr*1 gene. *Mol. Cell Biol.*, **6**, 4039-4044.

SKINNER, D.G. & LIESKOVSKY, G. (1984). Contemporary cystectomy with pelvic node dissection compared to preoperative radiation therapy plus cystectomy in the management of invasive bladder cancer. *J. Urol.*, **131**, 1067-1073.

STERNBERG, C., YAGODA, A., SCHER, H.I., WATSON, R.C., HERR, H.W., MORSE, M.J., SOGANI, P.C., VAUGHAN, E.D., BANDER, N., WEISELBERG, L.R., GELLER, N., HOLLANDER, P.S., LIPPERMAN, R., FAIR, W.R. & WHITMORE, W.F. (1988). MVAC (methotrexate, vinblastine, doxorubicin, cisplatin) for advanced transitional cell carcinoma of the urothelium. *J. Urol.*, **137**, 663-666.

TWENTYMAN, P.R., FOX, N.E., WRIGHT, K.A. & BLEEHEN, N.M. (1986). Derivation and preliminary characterisation of adriamycin resistant lines of human lung cancer cells. *Br. J. Cancer*, **53**, 529-537.

UICC. (1978). *TNM Classification of Malignant Tumors*, 3rd edn. International Union against Cancer: Geneva.

VAN DER BLIEK, A.M. & BORST, P. (1989). Multidrug resistance. *Adv. Cancer Res.*, **52**, 165-203.

VERRELLE, P., MEISSONNIER, F., FONCK, Y., FEILLEL, V., DIONET, C., KWAITKOWSKI, F., PLAGNE, R. & CHASSAGNE, J. (1991). Clinical relevance of immunohistochemical detection of multidrug resistance P-glycoprotein in breast carcinoma. *J. Natl Cancer Inst.*, **83**, 111-116.

WANG, A.M., DOYLE, M.V. & MARK, D.F. (1989). Quantitation of mRNA by the polymerase chain reaction. *Proc. Natl Acad. Sci. USA*, **86**, 9717-9721.

ZASTAWNY, R.L., SALVINO, R., CHEN, J., BENCHIMOL, S. & LING, V. (1993). The core promoter region of the P-glycoprotein gene is sufficient to confer differential responsiveness to wild-type and mutant p53. *Oncogene*, **8**, 1529-1535.


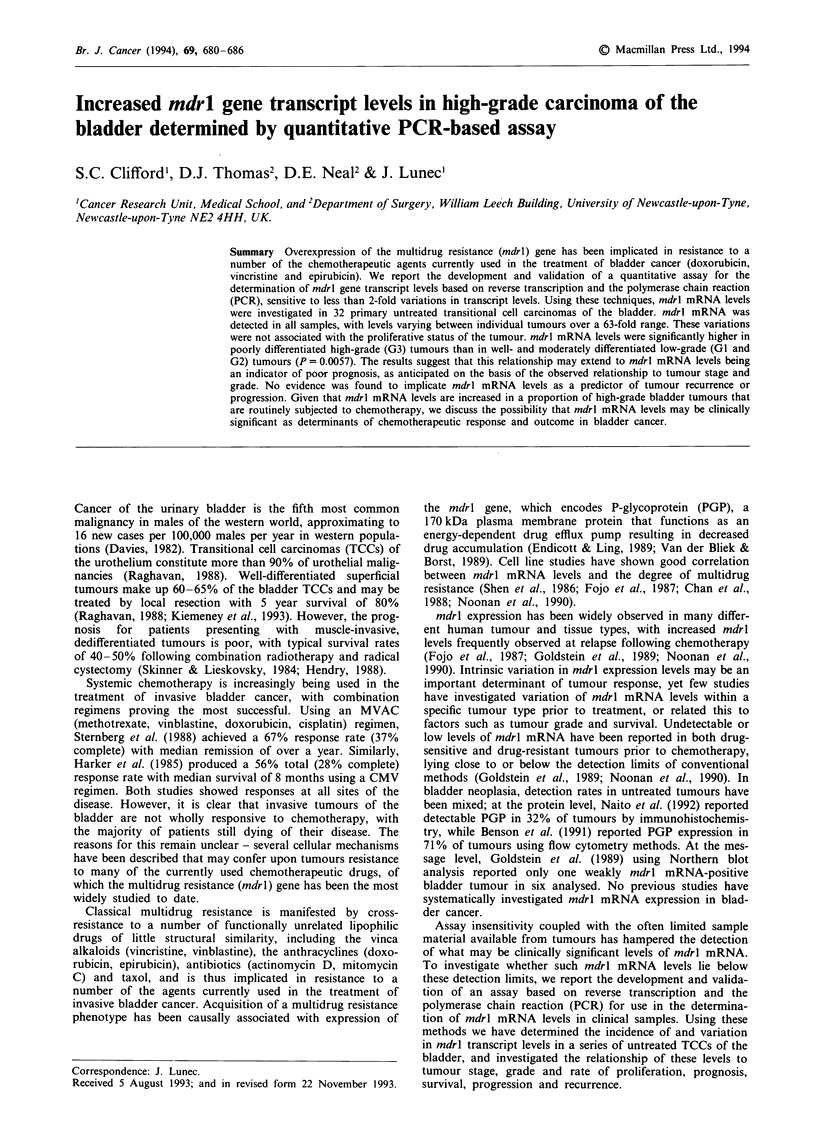

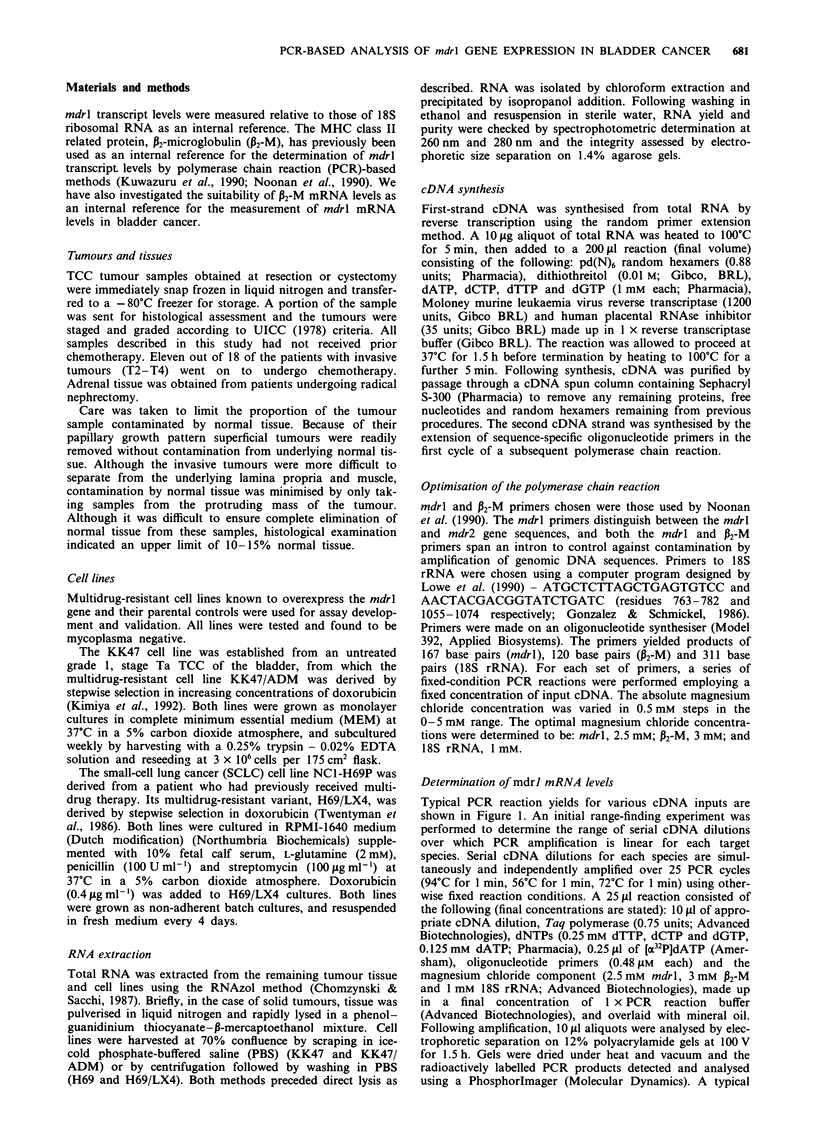

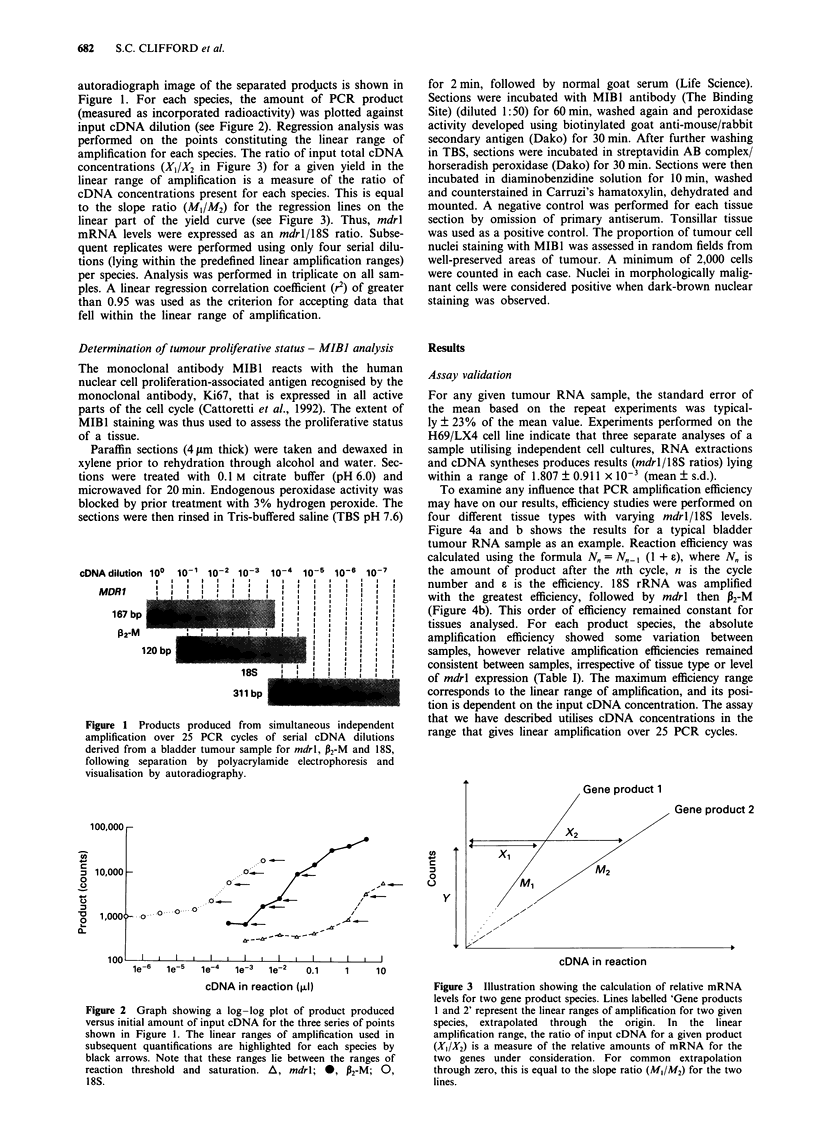

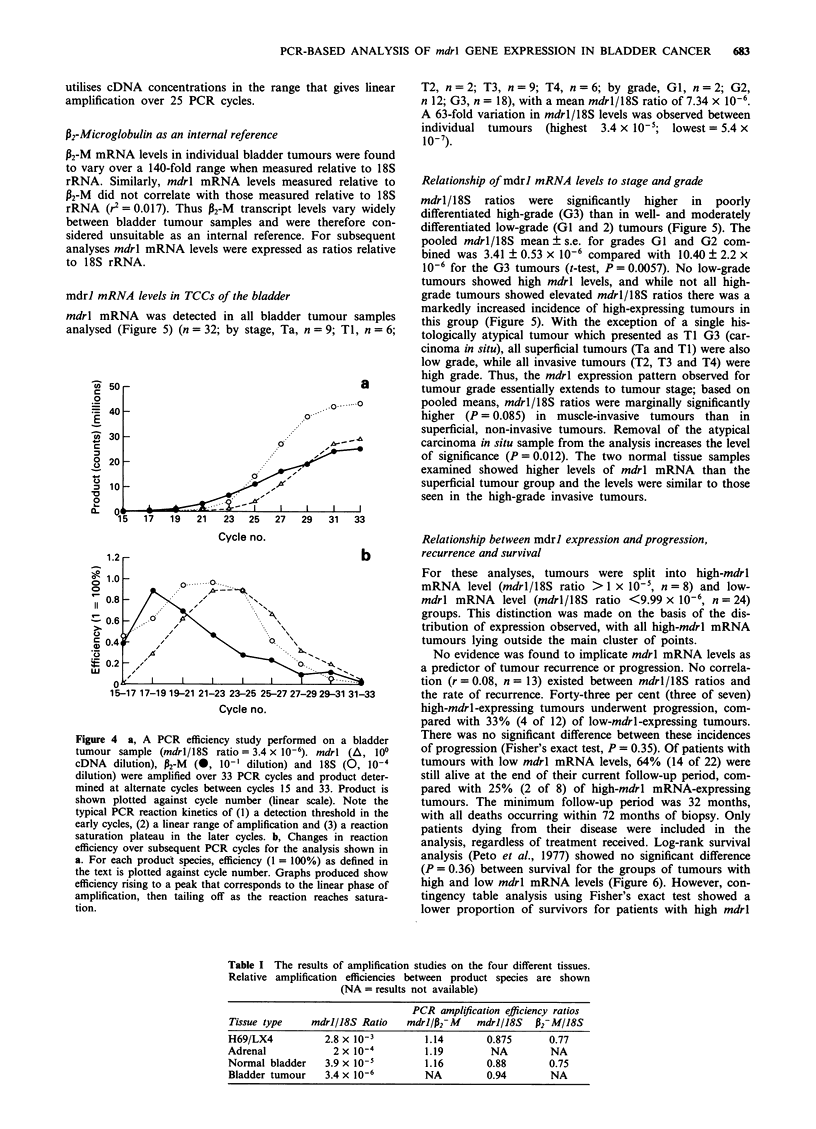

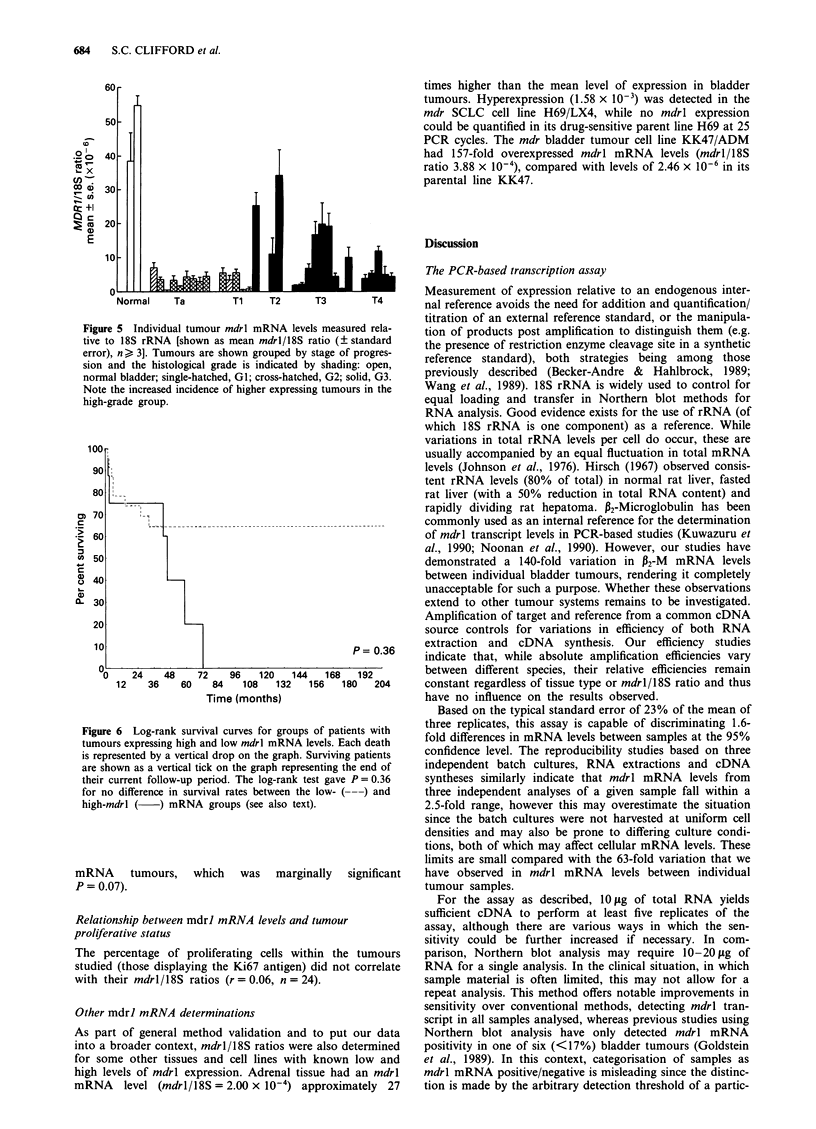

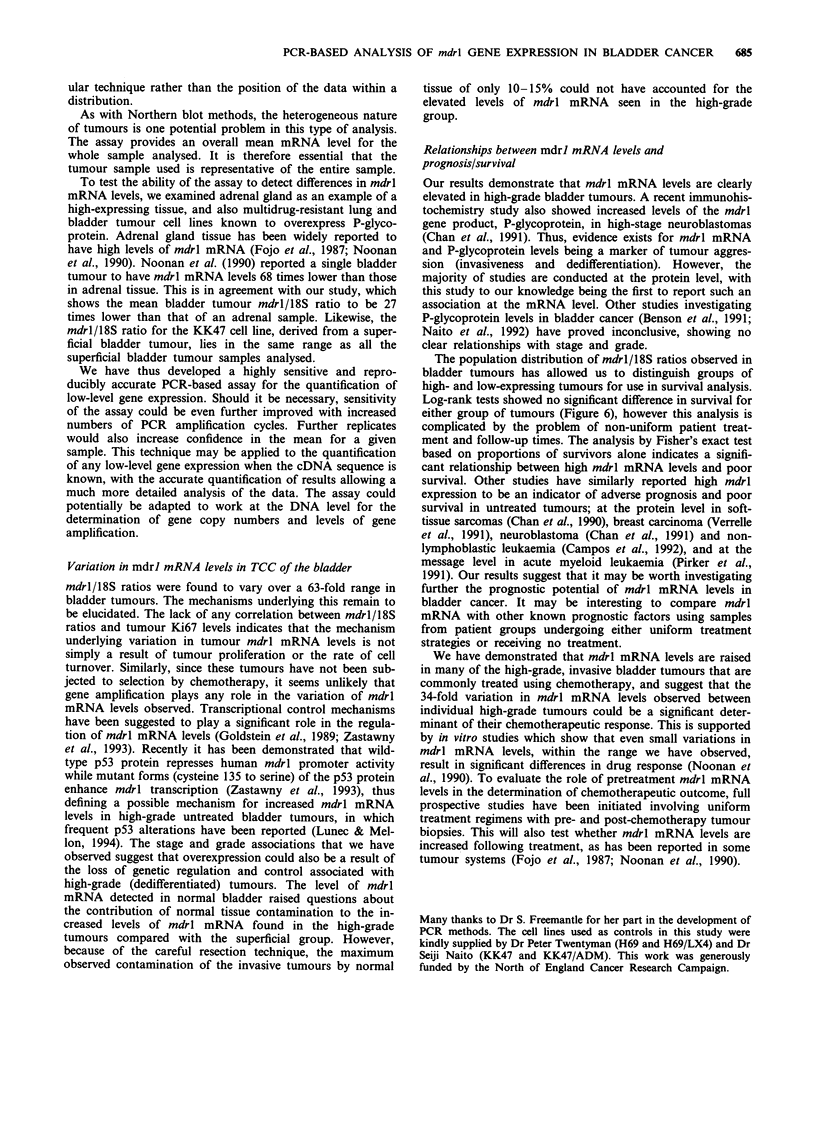

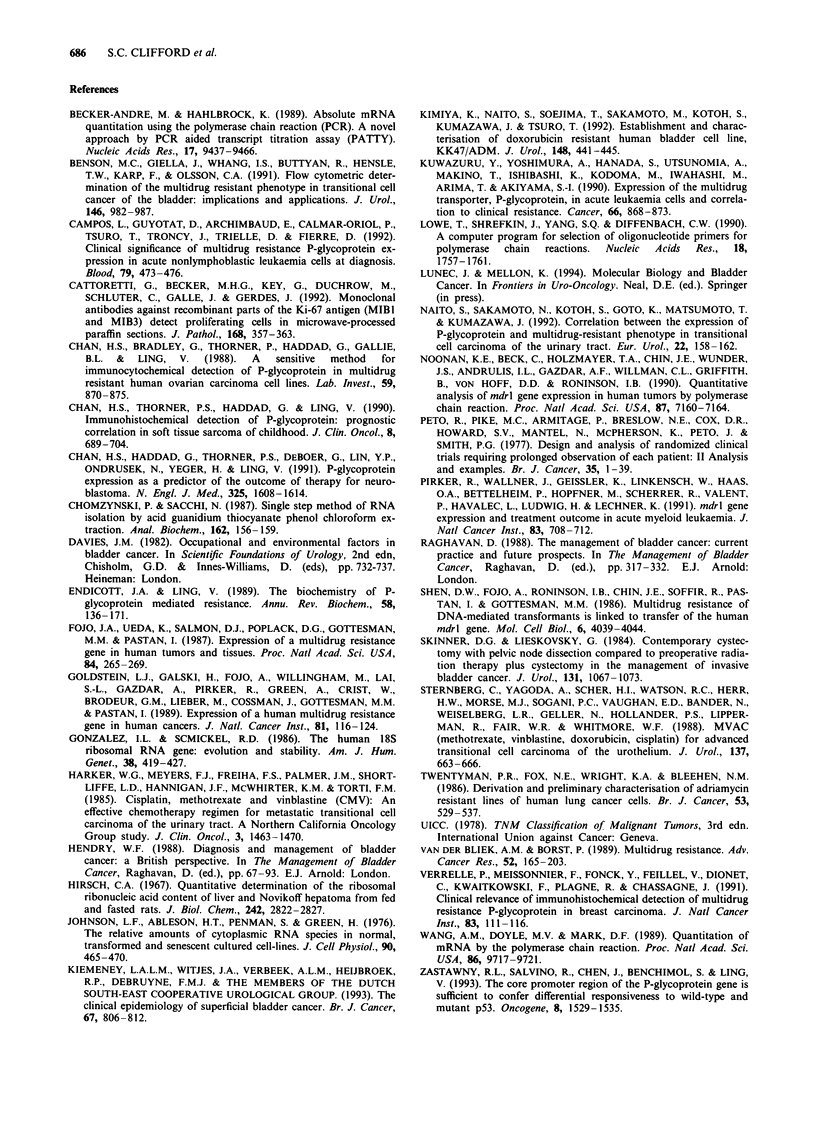


## References

[OCR_00985] Becker-André M., Hahlbrock K. (1989). Absolute mRNA quantification using the polymerase chain reaction (PCR). A novel approach by a PCR aided transcript titration assay (PATTY).. Nucleic Acids Res.

[OCR_00991] Benson M. C., Giella J., Whang I. S., Buttyan R., Hensle T. W., Karp F., Olsson C. A. (1991). Flow cytometric determination of the multidrug resistant phenotype in transitional cell cancer of the bladder: implications and applications.. J Urol.

[OCR_00998] Campos L., Guyotat D., Archimbaud E., Calmard-Oriol P., Tsuruo T., Troncy J., Treille D., Fiere D. (1992). Clinical significance of multidrug resistance P-glycoprotein expression on acute nonlymphoblastic leukemia cells at diagnosis.. Blood.

[OCR_01005] Cattoretti G., Becker M. H., Key G., Duchrow M., Schlüter C., Galle J., Gerdes J. (1992). Monoclonal antibodies against recombinant parts of the Ki-67 antigen (MIB 1 and MIB 3) detect proliferating cells in microwave-processed formalin-fixed paraffin sections.. J Pathol.

[OCR_01012] Chan H. S., Bradley G., Thorner P., Haddad G., Gallie B. L., Ling V. (1988). A sensitive method for immunocytochemical detection of P-glycoprotein in multidrug-resistant human ovarian carcinoma cell lines.. Lab Invest.

[OCR_01025] Chan H. S., Haddad G., Thorner P. S., DeBoer G., Lin Y. P., Ondrusek N., Yeger H., Ling V. (1991). P-glycoprotein expression as a predictor of the outcome of therapy for neuroblastoma.. N Engl J Med.

[OCR_01019] Chan H. S., Thorner P. S., Haddad G., Ling V. (1990). Immunohistochemical detection of P-glycoprotein: prognostic correlation in soft tissue sarcoma of childhood.. J Clin Oncol.

[OCR_01031] Chomczynski P., Sacchi N. (1987). Single-step method of RNA isolation by acid guanidinium thiocyanate-phenol-chloroform extraction.. Anal Biochem.

[OCR_01042] Endicott J. A., Ling V. (1989). The biochemistry of P-glycoprotein-mediated multidrug resistance.. Annu Rev Biochem.

[OCR_01047] Fojo A. T., Ueda K., Slamon D. J., Poplack D. G., Gottesman M. M., Pastan I. (1987). Expression of a multidrug-resistance gene in human tumors and tissues.. Proc Natl Acad Sci U S A.

[OCR_01053] Goldstein L. J., Galski H., Fojo A., Willingham M., Lai S. L., Gazdar A., Pirker R., Green A., Crist W., Brodeur G. M. (1989). Expression of a multidrug resistance gene in human cancers.. J Natl Cancer Inst.

[OCR_01060] Gonzalez I. L., Schmickel R. D. (1986). The human 18S ribosomal RNA gene: evolution and stability.. Am J Hum Genet.

[OCR_01067] Harker W. G., Meyers F. J., Freiha F. S., Palmer J. M., Shortliffe L. D., Hannigan J. F., McWhirter K. M., Torti F. M. (1985). Cisplatin, methotrexate, and vinblastine (CMV): an effective chemotherapy regimen for metastatic transitional cell carcinoma of the urinary tract. A Northern California Oncology Group study.. J Clin Oncol.

[OCR_01078] Hirsch C. A. (1967). Quantitative determination of the ribosomal ribonucleic acid content of liver and Novikoff hepatoma from fed and from fasted rats.. J Biol Chem.

[OCR_01083] Johnson L. F., Abelson H. T., Penman S., Green H. (1977). The relative amounts of the cytoplasmic RNA species in normal, transformed and senescent cultured cell lines.. J Cell Physiol.

[OCR_01089] Kiemeney L. A., Witjes J. A., Verbeek A. L., Heijbroek R. P., Debruyne F. M. (1993). The clinical epidemiology of superficial bladder cancer. Dutch South-East Cooperative Urological Group.. Br J Cancer.

[OCR_01096] Kimiya K., Naito S., Soejima T., Sakamoto N., Kotoh S., Kumazawa J., Tsuruo T. (1992). Establishment and characterization of doxorubicin-resistant human bladder cancer cell line, KK47/ADM.. J Urol.

[OCR_01102] Kuwazuru Y., Yoshimura A., Hanada S., Utsunomiya A., Makino T., Ishibashi K., Kodama M., Iwahashi M., Arima T., Akiyama S. (1990). Expression of the multidrug transporter, P-glycoprotein, in acute leukemia cells and correlation to clinical drug resistance.. Cancer.

[OCR_01109] Lowe T., Sharefkin J., Yang S. Q., Dieffenbach C. W. (1990). A computer program for selection of oligonucleotide primers for polymerase chain reactions.. Nucleic Acids Res.

[OCR_01120] Naito S., Sakamoto N., Kotoh S., Goto K., Matsumoto T., Kumazawa J. (1992). Correlation between the expression of P-glycoprotein and multidrug-resistant phenotype in transitional cell carcinoma of the urinary tract.. Eur Urol.

[OCR_01126] Noonan K. E., Beck C., Holzmayer T. A., Chin J. E., Wunder J. S., Andrulis I. L., Gazdar A. F., Willman C. L., Griffith B., Von Hoff D. D. (1990). Quantitative analysis of MDR1 (multidrug resistance) gene expression in human tumors by polymerase chain reaction.. Proc Natl Acad Sci U S A.

[OCR_01133] Peto R., Pike M. C., Armitage P., Breslow N. E., Cox D. R., Howard S. V., Mantel N., McPherson K., Peto J., Smith P. G. (1977). Design and analysis of randomized clinical trials requiring prolonged observation of each patient. II. analysis and examples.. Br J Cancer.

[OCR_01140] Pirker R., Wallner J., Geissler K., Linkesch W., Haas O. A., Bettelheim P., Hopfner M., Scherrer R., Valent P., Havelec L. (1991). MDR1 gene expression and treatment outcome in acute myeloid leukemia.. J Natl Cancer Inst.

[OCR_01155] Shen D. W., Fojo A., Roninson I. B., Chin J. E., Soffir R., Pastan I., Gottesman M. M. (1986). Multidrug resistance of DNA-mediated transformants is linked to transfer of the human mdr1 gene.. Mol Cell Biol.

[OCR_01174] Twentyman P. R., Fox N. E., Wright K. A., Bleehen N. M. (1986). Derivation and preliminary characterisation of adriamycin resistant lines of human lung cancer cells.. Br J Cancer.

[OCR_01188] Verrelle P., Meissonnier F., Fonck Y., Feillel V., Dionet C., Kwiatkowski F., Plagne R., Chassagne J. (1991). Clinical relevance of immunohistochemical detection of multidrug resistance P-glycoprotein in breast carcinoma.. J Natl Cancer Inst.

[OCR_01195] Wang A. M., Doyle M. V., Mark D. F. (1989). Quantitation of mRNA by the polymerase chain reaction.. Proc Natl Acad Sci U S A.

[OCR_01200] Zastawny R. L., Salvino R., Chen J., Benchimol S., Ling V. (1993). The core promoter region of the P-glycoprotein gene is sufficient to confer differential responsiveness to wild-type and mutant p53.. Oncogene.

[OCR_01184] van der Bliek A. M., Borst P. (1989). Multidrug resistance.. Adv Cancer Res.

